# Supplementary figures and images for: Bile acids target proteolipid nano-assemblies of EGFR and phosphatidic acid in the plasma membrane for stimulation of MAPK signaling
Source: PLoS One. 2018 Aug 31;13(8):e0198983. doi: 10.1371/journal.pone.0198983 (PMC6118352; doi:10.1371/journal.pone.0198983)

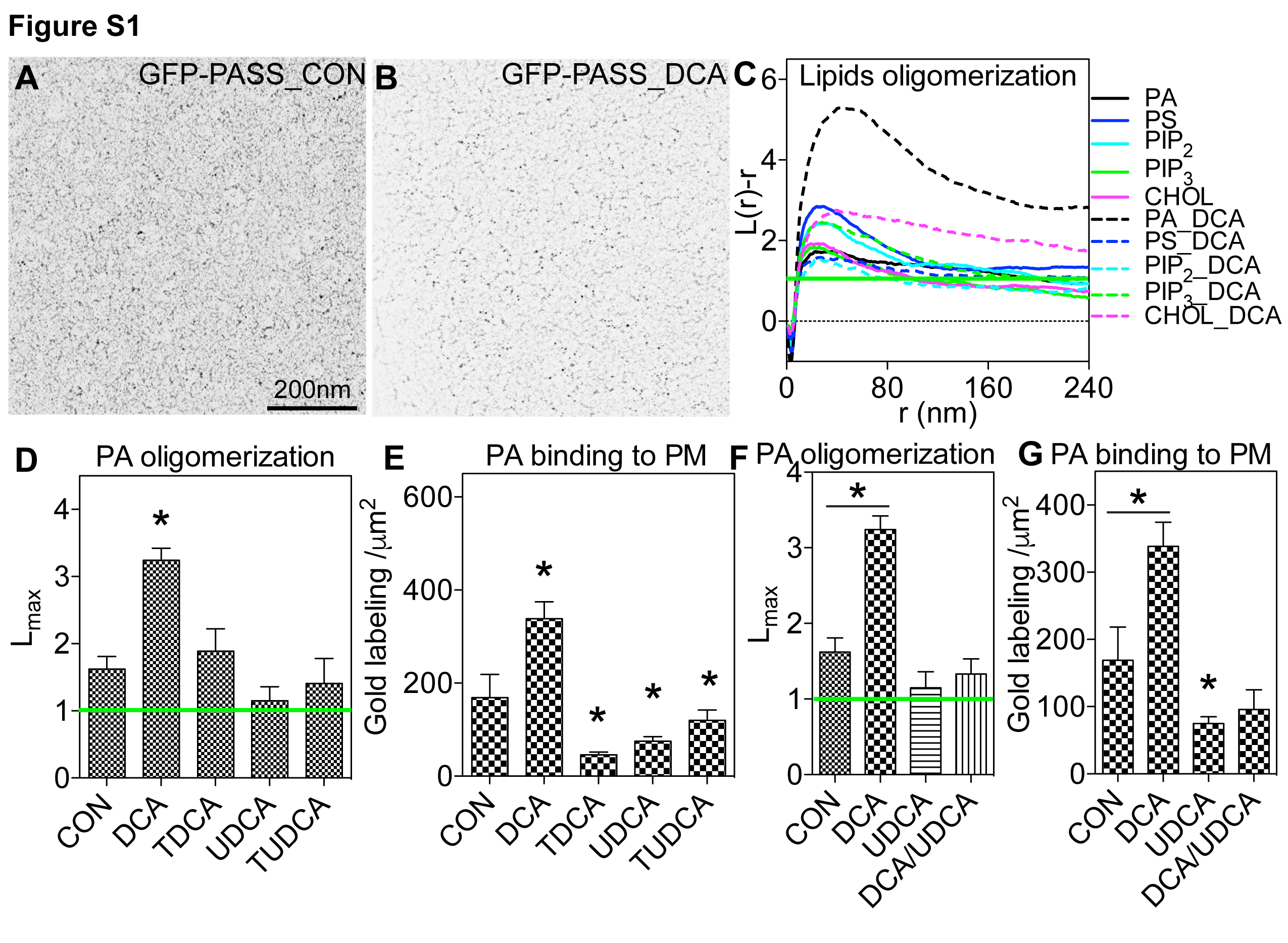

Supplement: S1 Fig — EM micrographs of intact basolateral PM of Caco-2 cells ectopically expressing GFP-PASS under control condition (A) or treated with 1μM DCA for 5 minutes (B). GFP-PASS bound to PA on the intact Caco-2 basal PM was immunolabeled with 4.5nm gold nanoparticles conjugated with anti-GFP antibody. (C) Univariate clustering of gold particles labeling various GFP-tagged lipid-binding domains without / with DCA (1μM, 5 minutes) was calculated using K-function analysis. Extent of clustering, L(r)-r, was plotted against length scale, r, with the peak value termed as Lmax. L(r)-r values above the 99% confidence interval (99%C.I.) of 1 indicate statistically meaningful clustering, whereas L(r)-r values below 99%C.I. indicate uniform distribution. Optimal clustering, Lmax, (D) and gold labeling (E) of GFP-PASS in Caco-2 cells treated with 1μM of various unconjugated and tauro-conjugated bile acids were quantified using EM-univariate clustering analysis. Optimal clustering (F) and PM binding (G) of GFP-PASS in Caco-2 cells exposed to DCA alone, UDCA alone or combination of both DCA and UDCA were quantified using EM-univariate spatial analysis. Statistical significance between untreated and DCA-treated conditions in all clustering analyses was evaluated using bootstrap tests, with * indicating p<0.05. Statistical significance between untreated and DCA-treated conditions in gold labeling was examined using one-way ANOVA, with * indicating p<0.05. (TIF) [file pone.0198983.s001.tif]

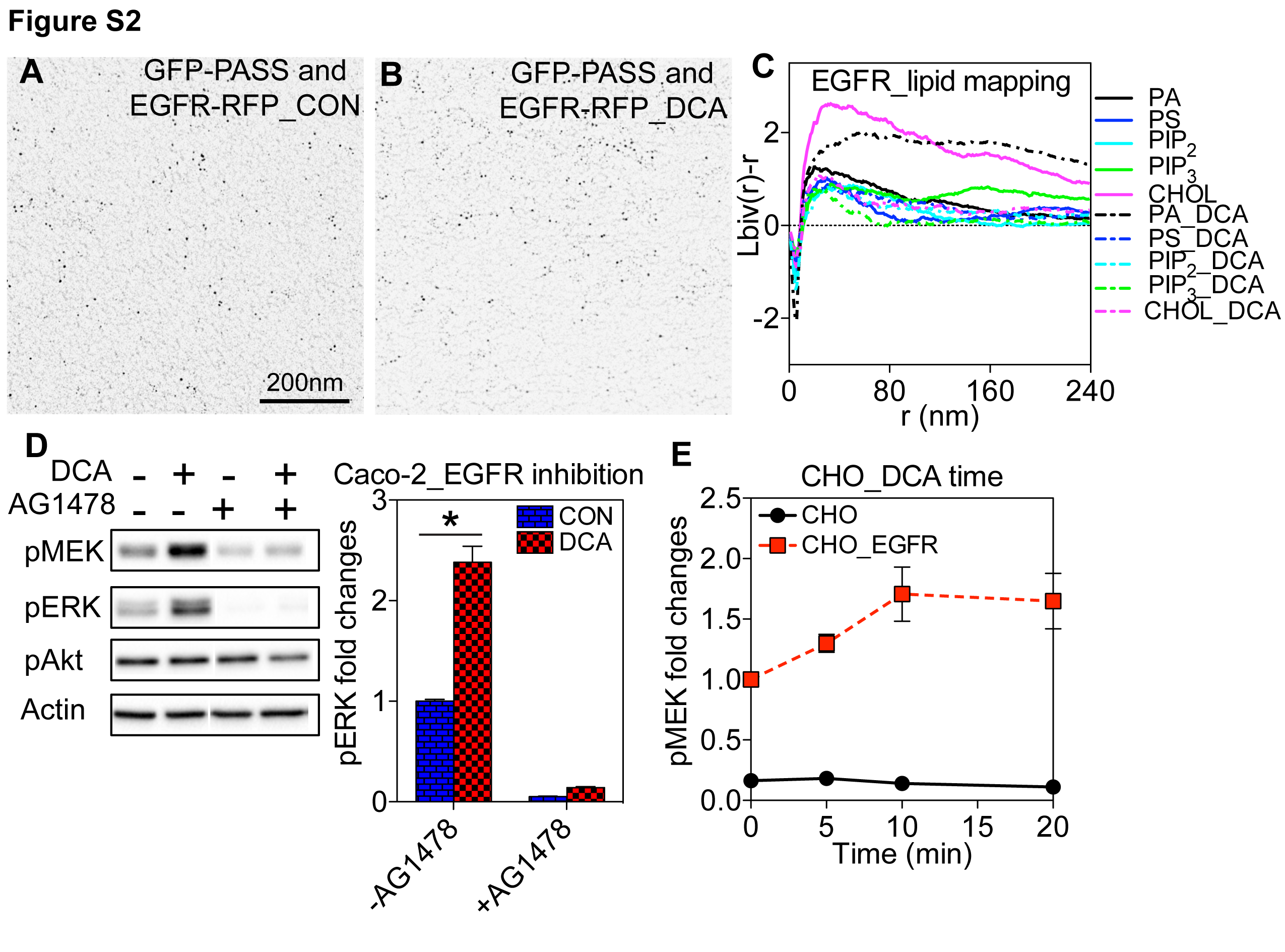

Supplement: S2 Fig — EM micrographs of intact basal PM of Caco-2 cells co-expressing GFP-PASS and EGFR-RFP untreated (A) or treated with 1μM DCA for 5 minutes (B). GFP-PASS was immunolabeled with 6nm gold conjugated to anti-GFP antibody, while EGFR-RFP was immunolabeled with 2nm gold coupled to anti-RFP antibody. (C) Bivariate K-function calculated the co-localization between 6nm and 2nm gold populations on intact Caco-2 basal PM sheets. Extent of co-localization, Lbiv(r)-r, was plotted against length scale, r. Lbiv(r)-r values above the 95%C.I. indicate statistically significant co-localization. Each Lbiv(r)-r curve was then integrated between r values of 10 and 110 to yield integrated L-bivariate, or LBI, to summarize the spatial data. Statistical significance between untreated and DCA-treated conditions in bivariate co-localization analyses was evaluated using bootstrap tests, with * indicating p<0.05. (D) Caco-2 cells grown to a monolayer were serum-starved for 2 hours and 30 minutes before treatment of 1μM EGFR specific inhibitor AG1478 for 25 minutes and a subsequent co-incubation with AG1478 and 1μM DCA for 5 minutes. Whole cell lysates were collected and blotted using antibodies against pMEK, pERK, or pAkt. (E) Wild-type CHO cells or CHO cells stably expressing EGFR-GFP were serum-starved for 10 minutes before incubation with 30μM DCA for various time points to ensure total serum starvation time is 30 minutes. Whole cell lysates were collected and blotted against pMEK. Statistical significance was evaluated using one-way ANOVA, with * indicating p<0.05. (TIF) [file pone.0198983.s002.tif]

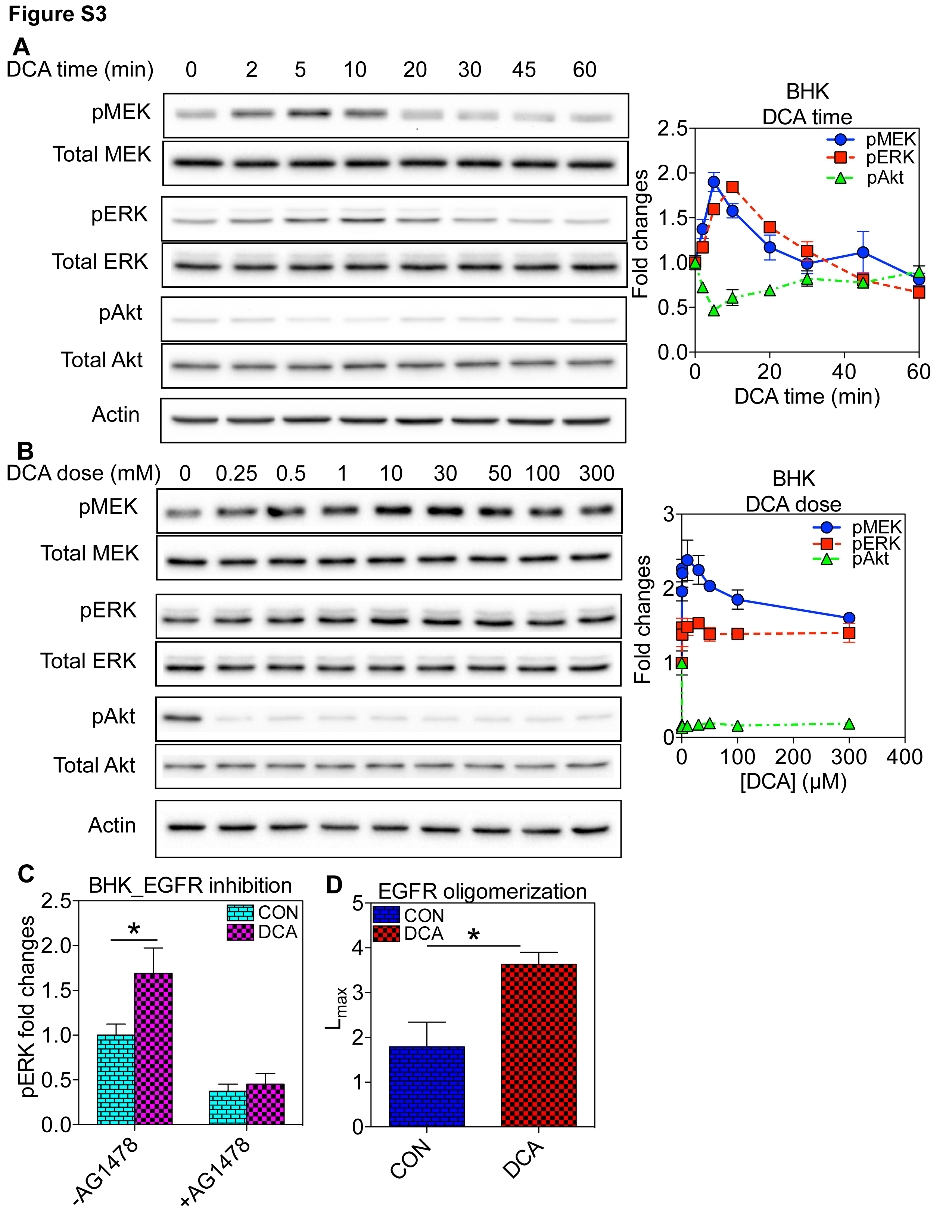

Supplement: S3 Fig — (A) BHK cells grown to 80–90% confluency were serum-starved for 1 hour before incubation with 30μM DCA for various time points to achieve total serum starvation time of 2 hours. Whole cell lysates were used to blot against pMEK, total MEK, pERK, total ERK, pAkt or total Akt, as well as the loading control actin. (B) BHK cells grown to 80–90% confluency were serum-starved for 1 hour and 55 minutes before incubation with various concentrations of DCA for 5 minutes. Whole cell lysates were used to blot against pMEK, total MEK, pERK, total ERK, pAkt or total Akt, as well as the loading control actin. (C) BHK cells were serum-starved for 1 hour and 30 minutes before treatment with 1μM AG1478 for 25 minutes and a subsequent co-incubation with AG1478 and 30μM DCA for 5 minutes. Whole cell lysates were used to blot against pMEK, pERK, or pAkt. Statistical significance was evaluated using one-way ANOVA, with * indicating p<0.05. (D) EM-univariate clustering experiment was conducted in BHK cells expressing EGFR-GFP without / with 30μM DCA. Intact apical PM sheets of non-polarized BHK cells were attached to EM grids and immunolabeled with 4.5nm gold particles conjugated to anti-GFP antibody. Univariate clustering of the gold particles was quantified using univariate K-function analysis. Lmax values indicate the extent of oligomerization of EGFR-GFP in apical PM of BHK cells. Statistical significance between untreated and DCA-treated conditions in univariate clustering analyses was evaluated using bootstrap tests, with * indicating p<0.05. (TIF) [file pone.0198983.s003.tif]
